# Econometric modelling of multiple self-reports of health states: The switch from EQ-5D-3L to EQ-5D-5L in evaluating drug therapies for rheumatoid arthritis^☆^

**DOI:** 10.1016/j.jhealeco.2017.06.013

**Published:** 2017-09

**Authors:** Mónica Hernández-Alava, Stephen Pudney

**Affiliations:** School of Health and Related Research, University of Sheffield, UK

**Keywords:** EQ-5D, Ordinal response, Copula, Mixture models, Rheumatoid arthritis, Mapping, Cost-effectiveness

## Abstract

EQ-5D is used in cost-effectiveness studies underlying many important health policy decisions. It comprises a survey instrument describing health states across five domains, and a system of utility values for each state. The original 3-level version of EQ-5D is being replaced with a more sensitive 5-level version but the consequences of this change are uncertain. We develop a multi-equation ordinal response model incorporating a copula specification with normal mixture marginals to analyse joint responses to EQ-5D-3L and EQ-5D-5L in a survey of people with rheumatic disease, and use it to generate mappings between the alternative descriptive systems. We revisit a major cost-effectiveness study of drug therapies for rheumatoid arthritis, mapping the original EQ-5D-3L measure onto a 5L valuation basis. Working within a comprehensive, flexible econometric framework, we find that use of simpler restricted specifications can make very large changes to cost-effectiveness estimates with serious implications for decision-making.

## Highlights

• We analyse joint responses to the original EQ-5D-3L and newer EQ-5D-5L instruments.
• Econometric modelling shows significant differences in mobility and pain domains.
• We develop flexible and efficient copula-mixture method of mapping from 3L to 5L.
• Switch to mapped EQ-5D-5L increases measured cost-effectiveness ratios substantially.

## 1 Introduction: EQ-5D-3L and EQ-5D-5L

The quality-adjusted life year (QALY) is one of the most widely used health benefit measures in economic evaluations of interventions, services or programmes designed to improve health. The QALY reflects concerns for both quality and length of life and allows health care decision makers to use a consistent approach across a broad range of disease areas, treatments, and patients. QALY estimation is based on patient-reported outcome measures (PROMs), of which EQ-5D is a leading example. EQ-5D is recommended by the English National Institute for Health and Care Excellence (NICE) for its technology appraisals, but it has wider international significance: public bodies in at least ten other countries also recommend EQ-5D as a basis for cost-effectiveness analysis.^1^ It is also increasingly used as a measure of performance in wider economic contexts, and as a generic health measure in population surveys (Devlin and Brooks, 2017). There is continuing debate about the basis of economic appraisal in health policy, with interest in wider outcome measures based on wellbeing or capabilities, income-variation valuations, and the use of weights for different aspects of disease such as burden of disease or rarity (Brazier and Tsuchiya, 2015). Nevertheless, for the foreseeable future, it seems inevitable that cost per QALY will continue to be the main driver of decisions in many public health services around the world.

EQ-5D measures patient outcomes across five dimensions: mobility, self-care, usual activities, pain/discomfort, and anxiety/depression. The original version of EQ-5D, which has been used in a large number of cost-effectiveness evaluations, measures each domain on a scale with three severity levels (no problems, some or moderate problems, extreme problems). Up to 3^5^ = 243 states of health can be described in this way, and each has been assigned a utility score on the basis of an analysis of preferences over length and quality of life using data from the general public (Dolan, 1997); full health is assigned a utility score of 1, 0 is equivalent to death, and negative values indicate health states worse than death.

Concerns about (lack of) sensitivity and floor/ceiling effects in the standard version recently led to the development of a new version, the EQ-5D-5L. The descriptive system covers the same five dimensions but the number of levels within each dimension has been extended from three to five (no problems, slight problems, moderate problems, severe problems, extreme problems). In addition, some of the wording has been modified to aid consistency and understanding.^2^ The maximum number of health states that can be described with the new version is 5^5^ = 3125. Several studies have reported better measurement properties in moving from the EQ-5D-3L to EQ-5D-5L in both specific patient and general population samples (Pickard et al., 2007, Janssen et al., 2013, Scalone et al., 2013, Agborsangaya et al., 2014, Jia et al., 2014). Utility value sets for EQ5D-5L have been proposed for England (Devlin et al., 2016), Japan (Ikeda et al., 2015), Canada (Xie et al., 2016), Uruguay (Augustovski et al., 2016), Netherlands (Versteegh et al., 2016) and Korea (Kim et al., 2016) and similar work is underway in many other countries. Many studies now include EQ-5D-5L instead of the standard version. Since these studies will form part of the evidence in future economic evaluations, it is important to assess the likely consequences for economic evaluation decisions of moving across the two different versions of EQ-5D, and to develop a basis for using the very large stock of existing evidence based on the 3L version.

If both variants of the EQ-5D instrument are observed in the same dataset and a utility score is available for each, it is possible to use a conditional statistical model to map directly from the 3L utility score to the 5L score or *vice versa*. However, that direct approach has three major disadvantages. First, utility scores have highly irregular empirical distributions and the most widely used mapping methods often fit poorly (Hernández-Alava et al., 2012). Second, use of a single utility score to summarise the 5-dimensional observed response fails to exploit all of the information contained in the observed EQ-5D responses. Third, the direct approach is necessarily specific to the particular scoring system used to construct utility values for the 3L and 5L health descriptions, making it hard to explore sensitivity to variations in the choice of scoring system. The alternative approach known as ‘response mapping’ (Gray et al., 2006) models the statistical relationship between the 3L and 5L responses and only brings utility scoring in at the final stage. By separating the logically distinct components of health state measurement and utility scoring, response mapping gives (in our view) a more natural way to proceed.

Although statistical mapping is often treated as a routine and arcane statistical task, it can have a critical impact on the outcome of economic decision-making, and the econometric assumptions used for mapping between alternative PROMs need to be examined very carefully. Those assumptions include: the choice of covariates for the mapping model, distributional specification, and independence or dependence of responses across the five domains of EQ-5D. Various statistical specifications appear in the small existing literature. Some authors have assumed conditional independence between the five domains of EQ-5D, estimating a separate model for each domain. Using this approach, van Hout et al. (2012) developed a mapping between EQ-5D-3L and EQ-5D-5L to construct an interim scoring system for EQ-5D-5L derived from the Dolan (1997) scores for EQ-5D-3L. However, independence is an implausible assumption: medical conditions may simultaneously affect multiple aspects of life – for instance severe pain may be accompanied by depression and curtailment of activities. Also, there may be individual-specific styles of questionnaire response which affect responses in all domains – some people tend to look on the bright side, while others do not. The conventional normality assumption built into the univariate or multivariate ordered probit model is also a strong one, and consistent estimation is not achieved in general if error distributions are non-normal, even if the model is correctly specified in all other respects. In Section 3 of the paper, we develop a multi-equation model that allows for the discrete EQ-5D response scales and uses a flexible mixture-copula specification of the error distributions. Importantly, we do not impose the assumption that responses in the five domains of EQ-5D are statistically independent. In Section 4, we apply the model to investigate the consistency of the responses to the two descriptive systems and the implied differences in the utility values. We derive the appropriate mapping technique in Section 5 and compare the results from mapping in both directions between the two variants of the EQ-5D instrument.

To explore the implications of modelling strategy for real-world policy decisions, we report an application to cost-effectiveness of treatments for rheumatoid arthritis (RA). We focus on RA partly for its inherent importance – among the 291 medical conditions covered by the 2010 Global Burden of Disease Study (Murray, 2012), RA ranked as the 42nd greatest contributor to global disability, measured in Years Lived with Disability (YLD), ranking immediately after malaria. It is also a rapidly growing problem; between 1990 and 2010, the estimated global burden of RA (adjusted for population growth and ageing) grew 15% in terms of YLD and 44% in terms of disability-adjusted life years (Cross et al., 2014). But data availability is another advantage; we have access to the National Data Bank for Rheumatic Diseases (NDB), which provides a unique RA-specific reference dataset that observes both versions of EQ-5D and also contains detailed clinical outcome measures. This allows us to explore one of the most important features of the mapping process, by varying the information provided by the covariates of the mapping model.

In Section 6, we re-visit the important CARDERA cost-effectiveness study (Choy et al., 2008, Wailoo et al., 2014) comparing four drug therapies for RA. We use statistical mapping to convert EQ-5D-3L responses into EQ-5D-5L QALYs, and find a large impact of the choice of statistical assumptions on the evaluation results. Our evidence suggests that the potential to move from EQ-5D-3L to EQ-5D-5L will pose significant methodological questions and may raise questions about some past decisions. We begin in Section 2 by describing the NDB data that we use for the EQ-5D-3L and EQ-5D-5L comparison – one of the few datasets available in which both variants of the instrument are carried in the same questionnaire.

## 2 The NDB dataset

The NDB is a register of patients with rheumatoid disease, primarily recruited by referral from US and Canadian rheumatologists. Information supplied by participants is validated by direct reference to records held by hospitals and physicians.^3^ Full details of the recruitment process are given by Wolfe and Michaud (2011). The EQ-5D responses and other patient-supplied data are collected by various means, primarily postal and web-based questionnaires completed directly by patients. Data collection began in 1998 and continues to the present, in waves administered in January and July of each year. In 2011, there was a switch from 3L to the 5L version of EQ-5D and both versions were collected in parallel during the January 2011 wave, to allow the effects of the switch to be accommodated in analyses spanning the whole period. Our principal aim is to use data from that wave of the survey to estimate a joint model of the 3- and 5L responses, which can then be used to map from 3- to 5L EQ-5D during the pre-2011 period and from 5- to 3L EQ-5D after January 2011. It then becomes possible to investigate the consistency of the two versions of EQ-5D and assess the impact of mapping between them.

### 2.1 EQ-5D response distributions

**Fig. 1:**
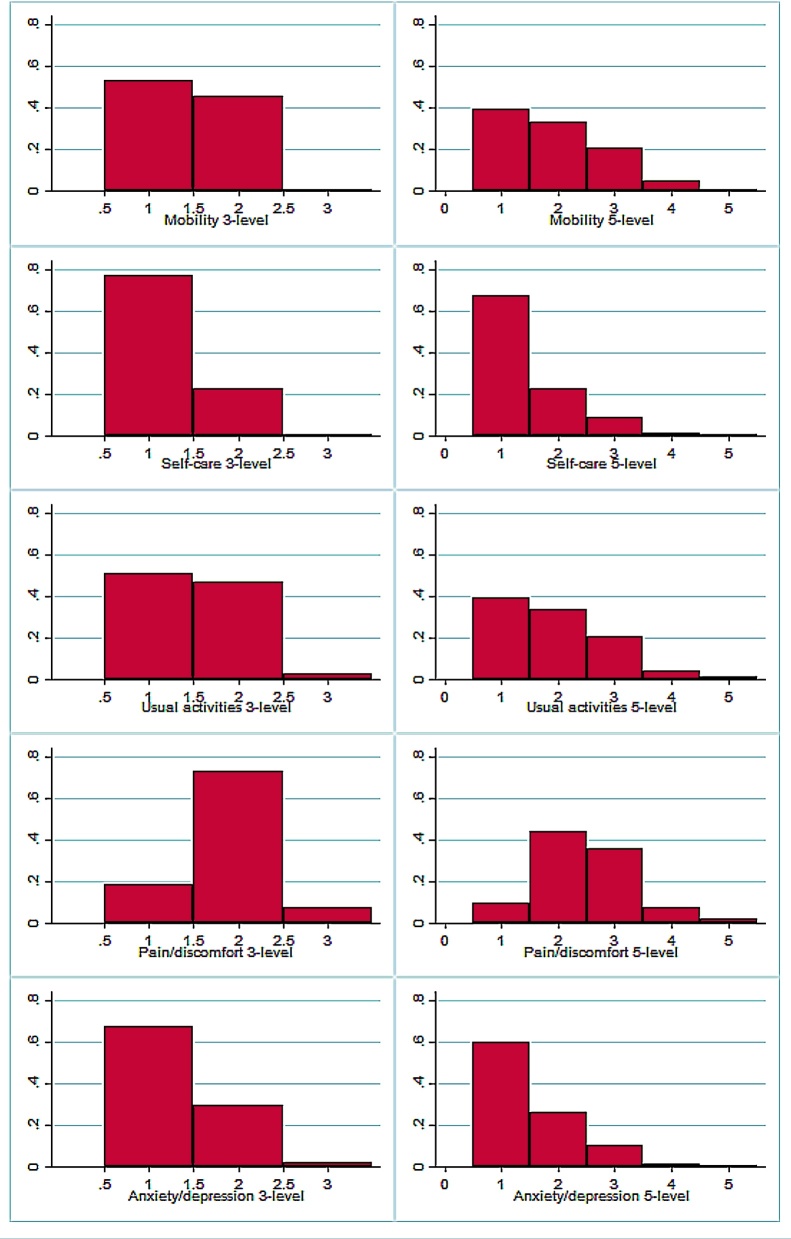
Response histograms for EQ-5D-3L and EQ-5D-5L (Jan 2011 wave of NDB, *n* = 5192).

Fig. 1 shows histograms of the NDB sample response distributions for the 3- and 5L versions of each domain of EQ-5D. There are clear differences between the distributional shapes for different domains: self-care and anxiety/depression have a dominant mode at the first category; the mobility and usual activities domains also have a decreasing profile but with a heavier central section, while the pain/discomfort domain shows a strong mode in the centre of the distribution. This variation in the shape of the component distributions underlines the need to use a suitably flexible model specification to analyse the relationship between variants of EQ-5D.

### 2.2 Utility scores

For each possible combination of EQ-5D responses, there is a utility value which allows overall health-related quality of life to be estimated and compared across individuals and conditions. We use the value sets produced by Dolan (1997) and Devlin et al. (2016) for the 3- and 5L versions of the instrument which, at present, are the standard choices for QALY measurement in England. Dolan (1997) used data from a representative sample of the UK population (2977 respondents). Each respondent valued 13 hypothetical health states using the time trade-off (TTO) method, generating valuations for a subsample of 42 of the 243 health states described by the EQ-5D-3L. The data were then modelled using regression methods to impute utility values for the remaining health states. Devlin et al. (2016) used a sample of the English population (996 respondents) who valued ten health states using a composite TTO approach, and seven paired comparisons of health states via discrete choice experiment tasks. The model selected for the EQ-5D-5L value set for England was a hybrid model using both sets of data (Feng et al., 2016).

**Fig. 2:**
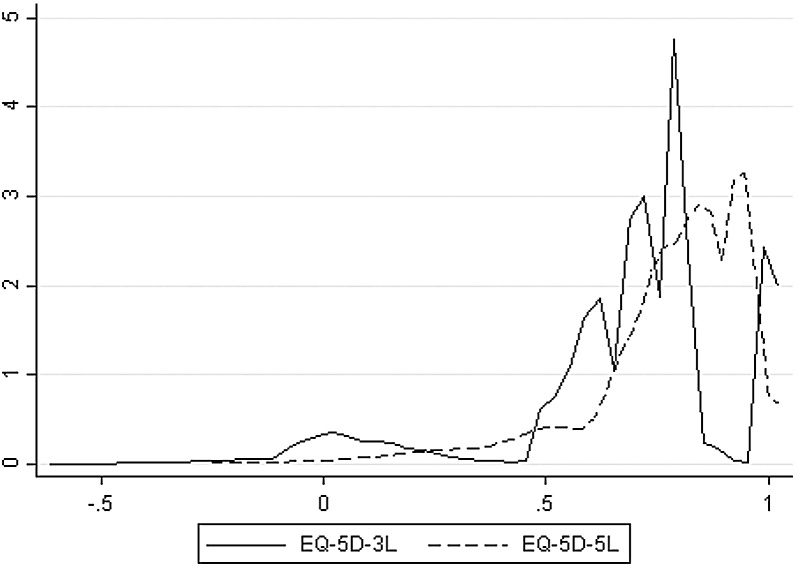
Smoothed empirical distributions of EQ-5D-3L and EQ-5D-5L (Jan 2011 wave of NDB, *n* = 5192).

Fig. 2 shows kernel density estimates of the distributions of utility scores in the NDB data, aggregated across all five domains. The distribution is smoother for the 5L version, particularly towards the top of the range, and this finer structure is a major reason for its adoption in practice. The distribution of utility scores for the 3L version of EQ-5D has two particularly worrying features. There are ranges with probability mass at or close to zero, particularly around 0.8–1.0 and 0.3–0.45. Consequently, methods for mapping to and from EQ-5D-3L which implicitly assume a smooth positive density can give very poor results (Hernández-Alava et al., 2012). The second striking feature of the distribution for EQ-5D-3L is the large group of cases with utility values close to zero, implying that a non-negligible proportion of patients with rheumatoid arthritis (RA) are in a state comparable to, or worse than, death. The outcomes of evaluation studies often rest on the ability of a therapy to improve quality of life for patients in very poor health, so the (perhaps implausibly) large frequency of such cases is a potential source of bias in NICE recommendations.

Table 1 summarises the January 2011 NDB data on the value scores for the two variants of EQ-5D in terms of their correlation with each other, with basic demographic characteristics, and with a set of clinical outcome measures. We use the Spearman rank correlation to show the strength of monotonic, not necessarily linear, associations, but the Pearson correlation shows a similar picture. There is a high correlation between the two variants of EQ-5D, but the 5L version has greater sensitivity, since correlations with demographics and clinical outcomes (in the lower panels of Table 1) are uniformly higher for EQ-5D-5L.

**Table 1:** Spearman correlations of 3- and 5L EQ-5D (Jan 2011 wave of NDB, *n* = 4856).

Variable	EQ-5D-3L	EQ-5D-5L
EQ-5D-3L	1.000	0.845
EQ-5D-5L	0.845	1.000

Female	−0.054	−0.074
Age	0.030	0.060

HAQ score (0–3)	−0.735	−0.758
Pain scale (0–10)	−0.707	−0.704
Overall RADAI score	−0.737	−0.746
Global severity (0–10)	−0.698	−0.721
Disease duration (months)	−0.057	−0.063
Fatigue scale (0–10)	−0.633	−0.669
Sleep disturbance scale (0–10)	−0.506	−0.541
Arthritis activity (general)	−0.611	−0.626
Arthritis activity (today)	−0.672	−0.673
RADAI joints (score)	−0.641	−0.648
RADAI joints (count)	−0.581	−0.589
Morning stiffness (0–6)	−0.538	−0.554
Co-morbidity index (0–9)	−0.344	−0.360
Physical component score (SF-6D)	0.727	0.700
Mental component score (SF-6D)	0.475	0.569
Health satisfaction (0–4)	−0.638	−0.671

Table 2 shows that there is a systematic difference in the 3L and 5L utility scores, with the old system generating utilities averaging (in the NDB data) only 87% of the utility values given by the new system. This alone could make a significant difference to some evaluation results. It would be inadvisible to address the issue with a simple proportional adjustment, since the ratio of mean scores is not constant but decreases as both general severity and pain increase, so the differences are minor at the top end of EQ-5D and much larger at the bottom. Table 2 gives means classified by levels of general disability (in three groups, scores 0–1, 1–2 and 2–3) and pain (in five groups 0–2, 2–4, 4–6, 6–8 and 8–10), as classified by the Stanford Health Assessment Questionnaire (HAQ). The HAQ is widely used by clinicians to measure treatment outcomes; see Bruce and Fries (2003) for a review.

**Table 2:** Means of EQ-5D-3L and EQ-5D-5L utility scores by severity of condition (Jan 2011 wave of NDB, *n* = 5192).

		3L	5L	Ratio
Overall		0.68	0.78	0.87
By general severity (HAQ) and pain scale category				
General^a^	Pain^b^	3L	5L	Ratio
1	1	0.87	0.92	0.95
1	2	0.76	0.86	0.89
1	3	0.72	0.83	0.87
1	4	0.67	0.78	0.87
1	5	0.51	0.72	0.71

2	1	0.74	0.81	0.91
2	2	0.66	0.76	0.87
2	3	0.60	0.73	0.82
2	4	0.52	0.64	0.81
2	5	0.30	0.53	0.56

3	1	0.63	0.71	0.89
3	2	0.54	0.65	0.83
3	3	0.45	0.57	0.79
3	4	0.35	0.48	0.73
3	5	0.15	0.35	0.43

a Groups corresponding to HAQ scores (1) [0–1); (2) [1–2) and (3) [2–3].

b Groups corresponding to pain scores (1) [0–2); (2) [2–4); (3) [4–6); (4) [6–8) and (5) [8–10].

Mapping from 3L to 5L involves two changes: a shift from the 3L health descriptive system to the 5L system, made using a predictive statistical mapping model; and a shift from the utility tariff developed for EQ-5D-3L to the utility tariff applicable to EQ-5D-5L. These two changes occur jointly, so it is not possible to disentangle fully the effect on cost-effectiveness calculations of mapping from the effect of the change in utility structure. However, within a fixed framework dictated by the given 3L and 5L utility tariffs, it is possible to compare the results produced by alternative specifications of the mapping model. This is our strategy, implemented within a comprehensive and flexible econometric approach.

## 3 A correlated copula model with mixture marginals

Our aim is to develop an econometric model of responses to the ten items of the 3L and 5L instruments. The specification is guided by six important considerations, intended to avoid unnecessarily strong restrictions on the data. The model should:

(i) Treat the 3L and 5L responses symmetrically so that it can be used for 3L → 5L and 5L → 3L mapping in a mutually consistent way.
(ii) Avoid the assumption that the 5L response scale is simply a more detailed categorisation than the 3L scale of the same underlying concept – structural differences between the two responses are permitted if empirically necessary.
(iii) Allow for the effects of covariates – here, age, sex and clinical outcome measures, without assuming that they necessarily influence 3L and 5L responses in the same way.
(iv) Capture the strong association between 3L and 5L responses within each health domain, without necessarily assuming that the strength of the association is the same in all parts of the health distribution – for example, someone who has experienced extreme pain may answer the pain questions in a more focused and coherent way than someone without experience of chronic pain. To achieve this, we use a copula approach (Trivedi and Zimmer, 2005) to specify the bivariate distribution of each 3L, 5L pair of responses.
(v) Be sufficiently flexible to fit the diverse response patterns shown in Fig. 1, so we generalise the usual assumption of normally-distributed errors by allowing for a 2-part normal mixture distribution, which can capture a wide range of distributional shapes.
(vi) Allow dependence across the five domains of EQ-5D, reflecting common underlying causes and individual-specific response styles; we achieve this by incorporating a random latent factor influencing responses in all domains.

In advance of the empirical analysis, there is no way of knowing which of these considerations is most important, so the resulting model is complex. Define 1 ≤ *Y*_3*id*_ ≤ 3 and 1 ≤ *Y*_5*id*_ ≤ 5 as the reported outcomes for the *d*th domain (*d* = 1, …, 5) of the 3- and 5L forms of EQ-5D. The model is a system of ten latent regressions, arranged in the five domain groups, with domain *d* containing the equations for *Y*_3*id*_ and *Y*_5*id*_:

(1) $$\left(\begin{array}{c} Y_{3\mathrm{id}}^{*}=X_{i}\beta_{3d}+U_{3\mathrm{id}}\\ Y_{5\mathrm{id}}^{*}=X_{i}\beta_{5d}+U_{5\mathrm{id}} \end{array}\right\},\;d=1,\ldots ,5$$

where *i* indexes independently sampled individuals, *X*_*i*_ is a collection of row vectors of covariates, *β*_3*d*_, *β*_5*d*_ are corresponding coefficient vectors and *U*_3*id*_, *U*_5*id*_ are unobserved errors which may be stochastically dependent and non-normal. The latent dependent variables $Y_{3\mathrm{id}}^{*},Y_{5\mathrm{id}}^{*}$ are not observed directly but they have observable ordinal counterparts, *Y*_3*id*_, *Y*_5*id*_, generated by the following threshold-crossing conditions:

(2) $$Y_{\mathrm{kid}}=q\;\mathrm{iff}\;\Gamma_{\mathrm{kqd}}\leq Y_{\mathrm{kid}}^{*}<\Gamma_{k(q+1)d};\;q=1,\ldots ,Q_{k};\;k=3,5$$

where *Q*_*k*_ = 3 or 5 is the number of categories of *Y*_*kid*_ and the Γ_*kqd*_ are threshold parameters, with Γ_*k*1*d*_ = −∞ and $\Gamma_{k(Q_{k}+1)d}=+\infty$.

High-dimensional ordinal-variable applications present major computational problems. Currently, there is only a single published model of EQ-5D responses that relaxes independence (Conigliani et al., 2015), using a 5-equation correlated multivariate ordered probit model to predict EQ-5D responses from aggregate SF12 scores. Using that model in our 10-dimensional 3L-5L mapping context would involve estimation of 45 residual covariance parameters, with a likelihood requiring numerical integration over a 10-dimensional rectangle. Past experience with similar maximum simulated likelihood problems, using best-practice simulation methods like Halton sequences, tells us that likelihood-based tests and fit statistics are not robust enough for model comparisons to be reliable. The conventional ordered probit model also involves normality assumptions that are critical to its consistency property and which we want to relax.

Possible solutions to the dimensionality problem work by imposing structure on the joint distribution of the latent $Y_{\mathrm{kid}}^{*}$. In the copula literature, the most common approach is to build it up from bivariate component distributions, often using vine structures (Bedford and Cooke, 2002, Panagiotelis et al., 2012). However, that is most convincing when there is a natural ordering of the observed variables, particularly temporal sequencing (as in the application by Panagiotelis et al., 2012 to a sequence of four observations on headache spaced through the day). In our case, although the component items of EQ-5D-5L were asked in sequence and then the items of EQ-5D-3L later in the questionnaire, that ordering does not correspond at all to the natural connections between the 3L and 5L items through their shared meaning. For that reason, we adopt a different approach, using five separate bivariate copulas for the five domains of EQ-5D, and connecting the domains via a latent factor *V* which represents common influences on the respondent's responses. The error *U*_*kid*_ is decomposed into the latent factor *V*_*i*_ and a specific error *ε*_*kid*_ correlated within but not between domains:

(3) $$U_{\mathrm{kid}}=\psi_{\mathrm{kd}}V_{i}+\varepsilon_{\mathrm{kid}}$$

where the *ψ*_*kd*_ are a set of ten parameters. We make the standard assumptions that, conditional on *X*_*i*_: *V*_*i*_ is independent of all the *ε*_*kid*_; the *ε*_*kid*_ are all mutually independent, except that *ε*_3*id*_, *ε*_5*id*_ are possibly dependent within any health domain *d*.

We use a copula representation to capture dependence between the 3L and 5L responses for any domain. Suppressing the *i* subscript, define *F*_*d*_(*ε*_3*d*_, *ε*_5*d*_) as the distribution function (df) for domain *d* and *F*_3*d*_(*ε*_3*d*_) = *F*_*d*_(*ε*_3*d*_, ∞) and *F*_5*d*_(*ε*_5*d*_) = *F*_*d*_(∞, *ε*_5*d*_) to be the marginals. Their joint df for domain *d* is specified as:

(4) $$F_{d}(\varepsilon_{3d},\varepsilon_{5d})=c_{d}(G_{3d}(\varepsilon_{3d}),G_{5d}(\varepsilon_{5d});\theta_{d})$$

where *G*_*kd*_(·) is the marginal df of *ε*_*kd*_ and *θ*_*d*_ is a parameter controlling the dependence between *ε*_3*d*_ and *ε*_5*d*_. The function *c*_*d*_(·) is known as a copula and, together with the marginals *G*_3*d*_(·), *G*_5*d*_(·) it uniquely characterises the bivariate distribution of *ε*_3*d*_, *ε*_5*d*_. It has the properties *c*_*d*_(0, *u*) = *c*_*d*_(*u*, 0) = 0 and *c*_*d*_(1, *u*) = *c*_*d*_(*u*, 1) = *u* for any 0 ≤ *u* ≤ 1 (Trivedi and Zimmer, 2005). We consider the following candidate forms:

$$\mathrm{Gaussian}:\;c(\varepsilon_{3},\varepsilon_{5})=\Phi (\Phi^{-1}(\varepsilon_{3}),\Phi^{-1}(\varepsilon_{5});\theta )$$

where Φ(., .;*θ*) is the distribution function of the bivariate normal with correlation coefficient −1 ≤ *θ* ≤ 1 and Φ^−1^(·) is the inverse of the univariate *N*(0, 1) df

$$\mathrm{Clayton}:\;c(\varepsilon_{3},\varepsilon_{5})=\left\{\begin{array}{cc} {\left[\max\left\{\varepsilon_{3}^{-\theta }+\varepsilon_{5}^{-\theta }-1,0\right\}\right]}^{-1/\theta } & \mathrm{for}\;0<\theta \leq \infty \\ \varepsilon_{3}\varepsilon_{5} & \mathrm{for}\;\theta =0 \end{array}\right)$$

$$\mathrm{Frank}:\;c(\varepsilon_{3},\varepsilon_{5})=\left\{\begin{array}{cc} -\frac{1}{\theta }\ln\left(1+\frac{\left(e^{-\theta \varepsilon_{3}}-1\right)\left(e^{-\theta \varepsilon_{5}}-1\right)}{e^{-\theta }-1}\right) & \mathrm{for}\;\theta \neq 0\\ \varepsilon_{3}\varepsilon_{5} & \mathrm{for}\;\theta =0 \end{array}\right)$$

$$\mathrm{Gumbel}:\;c(\varepsilon_{3},\varepsilon_{5})=\exp\left(-{\left[{\left(-\ln\varepsilon_{3}\right)}^{\theta }+{\left(-\ln\varepsilon_{5}\right)}^{\theta }\right]}^{1/\theta }\right)\;\mathrm{for}\;\theta \geq 1$$

$$\mathrm{Joe}:\;c(\varepsilon_{3},\varepsilon_{5})=1-{\left[{\left(1-\varepsilon_{3}\right)}^{\theta }+{\left(1-\varepsilon_{5}\right)}^{\theta }-{\left(1-\varepsilon_{3}\right)}^{\theta }{\left(1-\varepsilon_{5}\right)}^{\theta }\right]}^{1/\theta }\;\mathrm{for}\;\theta \geq 1$$

**Fig. 3:**
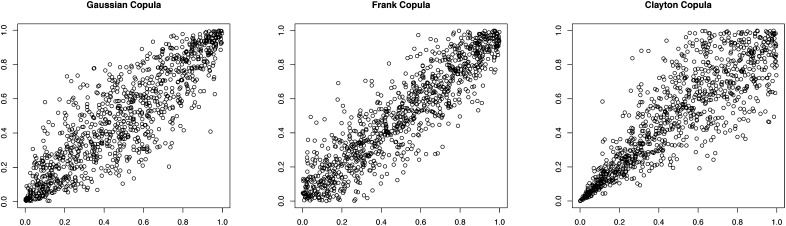
Pseudo-random samples drawn from three alternative copulas.

The Gaussian and Frank copulas are similar in that both allow for positive or negative dependence, symmetric in both tails, but the Frank form generates dependence weaker in the tails and stronger in the centre of the distribution. The Clayton copula allows only positive dependence, with strong left tail dependence and relatively weak right tail dependence; thus, if two variables are strongly correlated at low values but less so at high values, then the Clayton copula is a good choice. To show the effect of copula choice, Fig. 3 shows simulated scatter plots generated using these three copulas.^4^ The Gumbel and Joe copulas (not illustrated) display weak left tail dependence and strong right tail dependence, which is stronger for the Joe than the Gumbel copula.

The within-domain specification is completed by a normal mixture assumption which allows any of the errors *ε*_*kid*_ to have a non-normal form:

(5) $$G(\varepsilon )=\pi \Phi \left(\frac{\varepsilon -\mu_{1}}{\sigma_{1}}\right)+[1-\pi ]\Phi \left(\frac{\varepsilon -\mu_{2}}{\sigma_{2}}\right)$$

where 0 ≤ *π* ≤ 1 is the mixing parameter; (*μ*_1_, *μ*_2_) and (*σ*_1_, *σ*_2_ ≥ 0) are location and dispersion parameters constrained to satisfy the mean and variance normalizations *πμ*_1_ + (1 − *π*)*μ*_2_ ≡ 0 and $\pi (\sigma_{1}^{2}+\mu_{1}^{2})+(1-\pi )(\sigma_{2}^{2}+\mu_{2}^{2})=1$. These normal mixtures can capture a wide range of distributional shapes, including skewness and bimodality. The mixture (5) can be implemented with various degrees of generality, by assuming the same parameter values (*π*, *μ*_1_, *μ*_2_, *σ*_1_, *σ*_2_) for all error terms, or allowing them to vary with domain *d* = 1, …, 5 and/or EQ-5D design *k* = 3, 5. We specify a normal mixture distribution for the latent factor *V* also.

Conditional on *X*, the probability of observing any values *Y*_3*d*_ = *q* and *Y*_5*d*_ = *r* is:

(6) $$\begin{array}{cc} P(q,r|X,d) & =c_{d}(G_{\mathrm{kd}}(q+1),G_{\mathrm{kd}}(r+1))-c_{d}(G_{\mathrm{kd}}(q+1),G_{\mathrm{kd}}(r))\\ & \;-c_{d}(G_{\mathrm{kd}}(q),G_{\mathrm{kd}}(r+1))+c_{d}(G_{\mathrm{kd}}(q),G_{\mathrm{kd}}(r)) \end{array}$$

where *G*_*kd*_ denotes *G*_*kd*_(Γ_*kqd*_ − *Xβ*_*kd*_). The joint distribution of *Y*_31_, *Y*_51_, …, *Y*_35_, *Y*_55_ is:

(7) $$\mathrm{Pr}(Y_{31},Y_{51},\ldots ,Y_{35},Y_{55}|X)=\int \prod_{d=1}^{5}P(Y_{3d},Y_{5d}|X,v)\left[\frac{p}{s_{1}}\phi \left(\frac{v-m_{1}}{s_{1}}\right)+\frac{1-p}{s_{2}}\phi \left(\frac{v-m_{2}}{s_{2}}\right)\right]\mathrm{dv}$$

We use Gauss–Hermite quadrature with 15 integration points to evaluate the integral in (7) at each observation to give the likelihood function.

## 4 Modelling results

Our aim is to estimate the joint distribution of the responses to the 3L and 5L variants of the EQ-5D survey instrument, conditional on demographic characteristics (age and gender), and clinical measures of the severity of the underlying rheumatic condition. We use seven covariates: age, gender, the HAQ disability score, the pain scale, and the squares and product of the HAQ and pain scales.

The HAQ is based on patient self-reporting of the degree of difficulty experienced over the previous week in eight categories: dressing and grooming, arising, eating, walking, hygiene, reach, grip, and common daily activities. It is widely used by clinicians to measure health outcomes. It is scored in increments of 0.125 between 0 and 3 (although it is standard to consider it fully continuous), with higher scores representing greater degrees of functional disability. The HAQ instrument also includes separately a patient self-report of pain scored on a Visual Analogue Scale (0–10).

### 4.1 Domain-specific modelling

We start by examining each of the five domains of EQ-5D separately using a bivariate approach, implemented in the Hernández-Alava and Pudney (2016) Stata bicop routine. There are several reasons for this: it is computationally easier to make the choice of copula for each domain separately, and the process generates good parameter starting values for likelihood optimisation for the full model. Also, although conditional independence between domains is rather implausible, if independence is not rejected, or if it turns out to have little adverse impact on cost-effectiveness applications, then domain-specific modelling offers a simple and effective approach.

Table 3 summarises the sample fit of alternative copula functions for the 3L- and 5L variants for each of the five domains, where we retain the standard assumption of Gaussian marginals. There is no single best choice of copula: the Gaussian form fits best for dimensions 1 and 3 (mobility and usual activities), the Frank copula fits best for dimensions 2 and 5 (self-care and anxiety/depression) while the Gumbel copula fits best for the pain/discomfort dimension. This coincides with differences in the empirical distributions of Fig. 1 between these three groups of domains. The Frank copula (which allows weaker dependence in the tails than the centre of the distribution) works better than the Gaussian copula when the tails of the response distribution are relatively heavy. The Gumbel copula which has asymmetric dependence in the tails (stronger dependence at higher values) fits better when there is a central mode and implies different patterns of dependence in both tails of the distribution.

**Table 3:** Sample fit of domain-specific models for alternative copula functions with Gaussian marginals.

	Copula				
	Gaussian	Frank	Clayton	Gumbel	Joe
*Mobility domain*					
Log-likelihood	**−6656.54**	−6665.73	−6727.46	−6669.82	−6736.73
*χ*^2^(7) for *H*_0_: *β*_3_ = *β*_5_	29.02***	29.49***	23.82***	33.64***	37.14***

*Self-care domain*					
Log-likelihood	−4221.35	**-4212.35**	−4248.89	§	§
*χ*^2^(7) for *H*_0_: *β*_3_ = *β*_5_	8.31	5.98	5.35		

*Usual activities domain*					
Log-likelihood	**−6772.96**	−6796.04	−6866.11	−6785.64	−6829.65
*χ*^2^(7) for *H*_0_: *β*_3_ = *β*_5_	10.87	10.22	10.89	11.23	11.53

*Pain/discomfort domain*					
Log-likelihood	−6148.63	−6148.07	−6190.84	**−6147.80**	−6199.63
*χ*^2^(7) for *H*_0_: *β*_3_ = *β*_5_	29.75***	30.26***	32.71***	29.09***	26.82***

*Anxiety/depression domain*					
Log-likelihood	−6243.59	**−6238.86**	−6300.55	−6244.72	−6302.70
*χ*^2^(7) for *H*_0_: *β*_3_ = *β*_5_	12.05*	8.56	5.10	10.66	11.86

Best-fitting models in bold type (all models have 15 parameters).

* Statistical significance: 10%.

*** Statistical significance: 1%.

§ No convergence.

Table 3 also gives the results of the Wald test of the null hypothesis that the coefficient vectors relating the (latent) response to age, gender and disease severity are identical in the 3- and 5L variants. The hypothesis is clearly rejected for the domains of mobility and pain. This finding shows that the effect of the move to 5 levels is not simply a uniform re-alignment of the response level.^5^

The assumption of normal marginals for the errors *ε*_*kd*_ was acceptable in terms of the Akaike (AIC) and Bayesian (BIC) information criteria for the mobility, self-care and anxiety/depression domains, but there was significant evidence of modest departures from normality for the usual activities and pain/discomfort domains. Table 4 summarises the preferred specifications for those two domains, comparing them with the simpler Gaussian-marginal models. Note that the conclusions about the equality of coefficients are not affected by non-normality.

**Table 4:** Estimated non-normal error distributions.

Domain	Gaussian marginals		Non-Gaussian marginals			
	AIC	BIC	Preferred mixture specification	AIC	BIC	Coefficient equality test: *χ*^2^(7)
Usual activities^a^	13587.9	13725.5	Equal	13550.5	13707.8	8.39
Pain/discomfort^b^	12337.6	12475.3	Unequal	12252.9	12429.9	40.91***

a Gaussian copula.

b Gumbel copula.

*** Statistical significance: 1%.

**Fig. 4:**
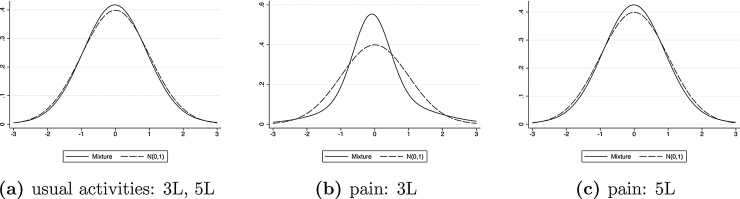
Estimated error distributions for the usual activities and pain/discomfort domain.

Fig. 4 plots the estimated distributions for the two domains where we find significant non-normality, and compares them to the *N*(0, 1) form. The distributions for the usual activities domain and for the EQ-5D-5L pain/anxiety domain are similar, both with a slightly fatter right tail of the distribution. The distribution for the EQ-5D-3L pain/anxiety dimension departs from normality with a much bigger central mode, consistent with its unique distributional shape in Fig. 1.

### 4.2 Joint modelling of all domains

We now examine the joint model. Table 5 summarises the sample fit of alternative joint models. All of them are based on the best fitting copulas for each dimension found in Section 4.1: Gaussian for mobility and usual activities; Frank for self-care and anxiety/depression; and Gumbel for pain/discomfort. Model (a) is the baseline model with no mixtures in *ε*; model (b) allows a common mixture, constrained to be the same for the errors in all ten equations; and model (c) allows for one common mixture for the usual activities domain and different mixtures for the 3L and 5L equations for pain/discomfort, following the pattern in Table 3. The joint log-likelihood, AIC and BIC for the model with independent EQ-5D dimensions are −29,958.431, 60,144.86 and 60,892.12 respectively, indicating that the joint model provides a better fit to the data. The joint model with a common mixture, model (b), gives the best fit to the data according to AIC and BIC. The conclusions about the equality of coefficients are not affected by the choice of error distributions and are in line with the conclusions of the domain-specific bivariate models. The estimated coefficients of the domain-specific bivariate and joint models are shown in Appendix Table A1.

**Table 5:** Sample fit of joint copula models.

	Type of mixture in *ε*		
	(a) None	(b) Equal	(c) Unequal
Log-likelihood	−29197.46	−29136.23	−29132.50
Number of parameters	115	118	124
AIC	58624.91	58508.46	58513.00
BIC	59378.73	59281.93	59325.80

Coefficient equality			
*Mobility domain*			
Equality of *β χ*^2^(7)	26.59***	26.53***	25.69***
Equality of *ψ χ*^2^(1)	0.18	0.29	0.00
Equality of *β* and *ψ χ*^2^(8)	28.59***	26.53***	28.73***

*Self-care domain*			
Equality of *β χ*^2^(7)	4.14	3.50	3.99
Equality of *ψ χ*^2^(1)	3.02*	3.37*	4.17**
Equality of *β* and *ψ χ*^2^(8)	9.60	8.91	10.80

*Usual activities domain*			
Equality of *β χ*^2^(7)	8.81	7.93	9.39
Equality of *ψ χ*^2^(1)	0.33	0.21	0.45
Equality of *β* and *ψ χ*^2^(8)	12.77	10.82	11.88

*Pain/discomfort domain*			
Equality of *β χ*^2^(7)	31.64***	30.19***	36.58***
Equality of *ψ χ*^2^(1)	18.80***	21.42***	29.27***
Equality of *β* and *ψ χ*^2^(8)	46.98***	50.65***	66.01***

*Anxiety/depression domain*			
Equality of *β χ*^2^(7)	9.27	8.70	9.36
Equality of *ψ χ*^2^(1)	2.68	2.75*	3.75*
Equality of *β* and *ψ χ*^2^(8)	11.07	10.54	11.99

* Statistical significance: 10%.

** Statistical significance: 5%.

*** Statistical significance: 1%.

**Fig. 5:**
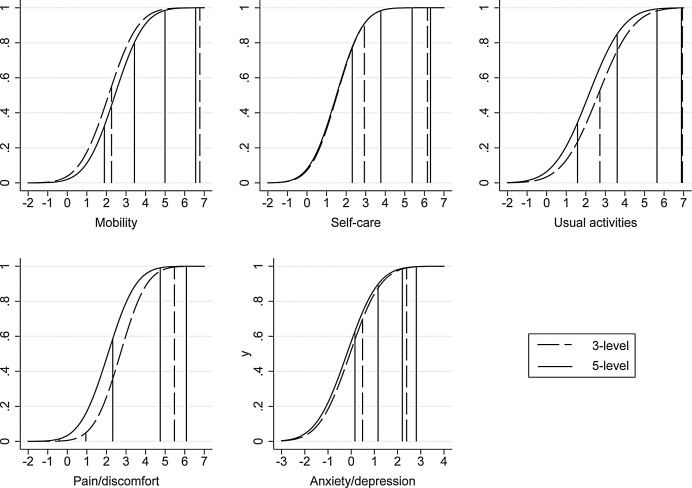
Estimated distribution functions and cutpoints for $Y_{3}^{*}$ and $Y_{5}^{*}$ (joint model, evaluated at covariate sample means).

Fig. 5 illustrates the effect of the differences in the distribution functions (df) of the latent variables $Y_{\mathrm{ikd}}^{*}$, evaluated at the sample mean values of the indexes $X_{i}{\hat{\beta }}_{\mathrm{kd}}$. These dfs calculated for the 3L and 5L equations are similar for the self-care, anxiety/depression and (to a lesser degree) usual activities domains. Moreover, the two threshold parameters for the 3L model fall respectively between the bottom two, and top two thresholds in the 5L model (${\hat{\Gamma }}_{52d}<{\hat{\Gamma }}_{32d}<{\hat{\Gamma }}_{53d}$ and ${\hat{\Gamma }}_{54d}<{\hat{\Gamma }}_{33d}<{\hat{\Gamma }}_{55d}$), which is consistent with the idea of a simple re-alignment of responses. However, for the mobility and pain/discomfort domains, the differences between dfs are sizeable and statistically significant, with the pain/discomfort domain displaying the largest difference. For both mobility and pain/discomfort, one of the 3L threshold parameters lies outside the range covered by the 5L threshold parameters, which is inconsistent with the simple realignment hypothesis.

## 5 Mapping

The best method of mapping between alternative preference-based measures depends on the nature of the cost-effectiveness study in which the measure is to be used. Suppose, for example, that the study is to be done on the new 5L basis, but the available evidence comes from a clinical trial in which the older EQ-5D-3L scale is measured. The key concept is the mean QALY, which should be constructed as *E*{*Q*(*υ*_5_(*Y*_5_))}, where *E*{ · } is the expectation with respect to whatever population is potentially affected by the treatment.

There are two technical issues to be considered in mapping from 3L evidence to 5L-based evaluation. First, the form of the function, *Q*(·), which maps utilities into QALYs. In most evaluation studies, the QALY calculation *Q*(·) is a linear function of the utilities, so that *E*{*Q*(*υ*_5_(*Y*_5_))} = *Q*(*E*{*υ*_5_(*Y*_5_)}). In other words, we can simply predict the utility outcome *υ*(*Y*_5_) and use that prediction in calculating QALYs. If the predictor is an unbiased (or consistent) estimator of *E*[*υ*(*Y*_5_)], it will give an unbiased (consistent) evaluation of the expected QALY.

The second issue is the choice of predictor for *υ*(*Y*_5_). We have argued here that a predictor based on a full model of *Pr*(*Y*_5_|*Y*_3_, *X*) uses more information and is capable of giving better results than the alternative approach to mapping, which attempts to model *E*(*υ*_5_(*Y*_5_)|*υ*_3_(*Y*_3_), *X*) directly – often using methods like linear regression which are not well suited to the non-standard distributions involved. When using our approach, it is important to realise that the utility scales *υ*(·) are nonlinear functions of the vector *Y*, so *E*(*υ*_5_(*Y*_5_)) ≠ *υ*_5_(*E*[*Y*_5_]). We should not map the observed 3L health description *Y*_3_ into the 5L descriptive system *Y*_5_ and then apply the utility scale *υ*_5_(·). Instead, the appropriate method is to use the model estimated from NDB data to evaluate the probability of each possible configuration of *Y*_5_ conditional on *Y*_3_, *X* and use those probabilities as weights to evaluate the conditional expectation of *υ*. The conditional df of the valuation *υ*_5_ is:

(8) $$\mathrm{Pr}(\upsilon_{5}(Y_{5})\leq ϒ|Y_{3},X)=\sum_{Y_{5}\in U_{ϒ}}\mathrm{Pr}(Y_{5}|Y_{3},X)$$

where *U*_ϒ_ is the set {*Y*_5_:*υ*_5_(*Y*_5_) ≤ ϒ} and ϒ is any given constant. The mean is:

(9) $$E(\upsilon_{5}(Y_{5})|Y_{3},X)=\sum_{Y_{5}\in S_{5}}\upsilon_{5}(Y_{5})\mathrm{Pr}(Y_{5}|Y_{3},X)$$

where *S*_5_ is the set of 3125 possible values that the vector *Y*_5_ might take.^6^

The choice of covariates is critical here. Mapping from *Y*_3_ rather than direct observation of *υ*_5_(*Y*_5_) introduces no bias in the calculation of mean QALYs if the conditional mean function *E*(*υ*_5_(*Y*_5_)|*Y*_3_, *X*) in the population represented by the reference sample used for mapping is identical to *E*(*υ*_5_(*Y*_5_)|*Y*_3_, *X*) in the population represented by the trial subjects. In general, reference samples and trial samples are drawn in quite different ways, and there is always a possibility that the statistical relationship between *Y*_3_ and *Y*_5_ could differ substantially between the two populations, leading to mapping bias. The use of covariates can reduce this risk by allowing for factors which might cause the *Y*_3_, *Y*_5_ association to differ across samples. Thus, even if *E*(*υ*_5_(*Y*_5_)|*Y*_3_) differs between the reference and trial samples, *E*(*υ*_5_(*Y*_5_)|*Y*_3_, *X*) may not, for a judicious choice of covariates. We explore this in the next section.

Several authors have commented on the loss of variation induced by mapping (Brazier et al., 2010, Longworth and Rowen, 2011, Fayers and Hays, 2014). The sample variance of the mean predictor (9) will always be lower than the variance of the unknown true *υ*_5_(*Y*_5_), because the modelling process can only predict variation in *υ*_5_(*Y*_5_) arising from *Y*_3_ and *X*, not the other “unexplained” components of variation. In standard cases where the QALY calculation is linear in utilities, this does not matter, since only the conditional mean of *υ*_5_(*Y*_5_) is required. If the aim were to estimate the variance of *υ*_5_(*Y*_5_), one would not do it by using the variance of the predictor (9); instead, the appropriate method is to calculate directly the variance of the distribution (8), which gives a consistent estimate of *var*(*υ*_5_(*Y*_5_)) if the mapping model is correctly specified and estimated.

**Fig. 6:**
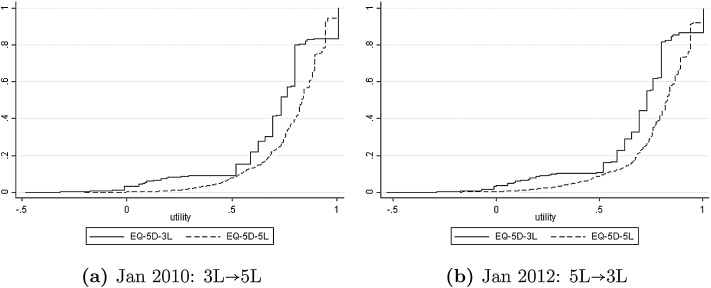
Cross-mapping based on independent domain-specific bivariate models.

**Fig. 7:**
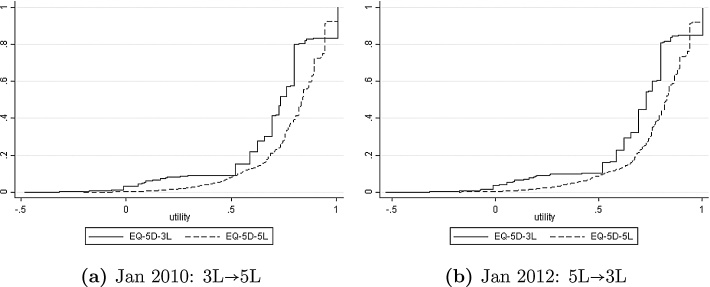
Cross-mapping based on the joint model with between-domain correlation.

If we evaluate (8) and (9) at each observation *Y*_*i*3_, *X*_*i*_, and then average over the sample, the result is a consistent estimator of the distribution of *υ*_5_(*Y*_5_) or its mean *E*[*υ*_5_(*Y*_5_)]. This can be done empirically for the pre-January 2011 waves of the NDB dataset and in reverse (predicting *Y*_3_ conditional on *Y*_5_) for the post-January 2011 waves. Fig. 6a uses the set of domain-specific bivariate models (assuming independence across domains) to compare the predictive df $n^{-1}\sum_{i=1}^{n}\mathrm{Pr}(\upsilon_{5}(Y_{5})\leq ϒ|Y_{i3},X_{i})$ and the directly-observed empirical df $n^{-1}\sum_{i=1}^{n}1(\upsilon_{3}(Y_{i3})\leq ϒ)$ for the Jan 2010 wave of NDB, where 1(·) is the indicator function. Fig. 6b makes the reverse comparison of the predictive df for *υ*_3_(*Y*_3_) with the empirical df of *υ*_5_(*Y*_5_) for the Jan 2012 wave. Fig. 7 makes the same comparisons for the joint model allowing for between-domain correlation.

There are two striking features of Fig. 6, Fig. 7, with important implications for the economic evaluations carried out for public bodies like NICE. First, the predictive and actual distributions of the 5L variant of EQ-5D are similar and much smoother than the corresponding distributions for the 3L variant. This is an encouraging finding: if a decision maker elects to recommend the use of the new 5L instrument and associated scoring, it may be possible to continue to use older 3L-based evidence with appropriate mapping to 5L. Second, there is a large difference between the 3L and 5L distributions of EQ-5D scores, whether directly observed or mapped. Utility scores tend to be systematically higher under the 5L scoring scheme, so the df for EQ-5D-3L lies entirely to the left of the df for EQ-5D-5L. If no other adjustment were made, this alone might be enough to change many evaluation results, in the absence of offsetting adjustments to the evaluation methodology.

Table 6 shows average values of directly-measured *υ*_3_(*Y*_3_) and the prediction *E*[*υ*_5_(*Y*_5_)|*Y*_3_, *X*] for the 2010 wave of NDB, and of the prediction *E*[*υ*_3_(*Y*_3_)|*Y*_5_, *X*] and directly-measured *υ*_5_(*Y*_5_) for the 2012 wave using the joint model. Results are given for the whole sample and subgroups defined in terms of disease severity and demographic characteristics; sample standard deviations of the measured and predicted utilities are also shown. As expected, there are higher mean values and smaller standard deviations for the EQ-5D-5L scores (whether predicted or directly observed) than for EQ-5D-3L, resulting from the different scoring of poor health states by the two value sets. Another consequence of this is the much steeper severity gradient for the mean EQ-5D-3L utilities than for EQ-5D.

**Table 6:** Means and standard deviations of actual and predicted (joint model) EQ-5D-3L and EQ-5D-5L by severity of condition, age and gender (NDB. January 2010 wave *n* = 3877; January 2012 wave *n* = 3911).

	January 2010		January 2012	
	EQ-5D-3L	EQ-5D-5L	EQ-5D-3L	EQ-5D-5L
	(actual)	(predicted)	(predicted)	(actual)
	Mean (SD)	Mean (SD)	Mean (SD)	Mean (SD)
Overall	0.70	0.79	0.69	0.78
	(0.25)	(0.16)	(0.21)	(0.19)

*Severity group*				
Mild	0.88	0.92	0.87	0.92
(HAQ group 1, Pain group 1)	(0.12)	(0.04)	(0.08)	(0.07)
Medium	0.62	0.71	0.61	0.73
(HAQ group 2, Pain group 3)	(0.15)	(0.09)	(0.11)	(0.11)
Severe	0.12	0.38	0.12	0.30
(HAQ group 3, Pain group 5)	(0.29)	(0.16)	(0.19)	(0.23)

Female <65	0.69	0.78	0.68	0.77
	(0.26)	(0.17)	(0.23)	(0.20)
Male<65	0.71	0.80	0.67	0.77
	(0.25)	(0.16)	(0.24)	(0.21)
Female 65–79	0.71	0.79	0.69	0.79
	(0.24)	(0.15)	(0.20)	(0.18)
Male 65–79	0.73	0.82	0.73	0.83
	(0.22)	(0.14)	(0.18)	(0.14)
Female ≥ 80	0.65	0.76	0.66	0.76
	(0.25)	(0.17)	(0.20)	(0.18)
Male ≥ 80	0.74	0.83	0.70	0.80
	(0.17)	(0.12)	(0.17)	(0.16)

There is a slight tendency for both the 3L and 5L utilities to decline over time as the health states of those individuals who appear in both waves tend to worsen. However, the means of predicted and directly-observed versions of each measure are remarkably close both overall and in terms of their severity and demographic profiles.

We also see the anticipated smaller standard deviations of the predicted than directly-observed utilities as a consequence of the use of expected value prediction. This is of no importance for the evaluation described in the next section (since the criterion is based on the mean QALY), but it would be a concern for any evaluation that aims to investigate the distributional pattern of QALY gains within each population group. In that case, appropriate measures constructed from the full distribution (8) would need to be used.

## 6 The impact on cost-effectiveness analysis

We now use a published cost-effectiveness study to examine the potential consequences of moving from EQ-5D-3L to EQ-5D-5L as a basis for economic evaluation. We first replicate the economic evaluation results in Wailoo et al. (2014), which use EQ-5D-3L data collected as part of a trial. Then we repeat the analysis using EQ-5D-5L obtained using the mapping models developed in this paper. Wailoo et al. (2014) estimate the cost-effectiveness of combinations of disease-modifying anti-rheumatic drugs (DMARDs) and short-term administration of the steroid prednisolone (PNS), using data from the 2-year CARDERA trial which involved 467 adult patients with early active RA (less than two years of disease duration) in a placebo-controlled factorial design. Two DMARDS were used in the trial, methotrexate (MTX) and ciclosporin (CS). All patients received MTX, half received step-down PNS^7^ and half CS, generating four treatment groups: (1) monotherapy (MTX only), (2) combination DMARDs (MTX and CS), (3) DMARD and steroid (MTX and PNS) and (4) triple therapy (MTX, CS and PNS). Further details of the methods and clinical effectiveness can be found in Choy et al. (2008).

The key criterion used in cost-effectiveness analysis is the Incremental Cost-Effectiveness Ratio (ICER), defined as the difference in costs between two different treatment strategies, expressed as a ratio to the difference in the QALYs that they achieve. Treatments with ICERs below a certain threshold are usually considered cost-effective. In the UK, NICE guidance on technology appraisal refers to a specific range £20,000–£30,000 (NICE, 2013), but see also Claxton et al. (2015) who argue for a lower threshold.

Resource use (prescription drugs, hospitalizations, tests, imaging, surgical procedures and community care visits) was directly observed over the two years of the trial and costed using 2011–2012 figures. The mean discounted cost of each treatment strategy is shown in the first row of Table 7, based on the sample of patients with complete data (*n* = 241). QALY estimates were derived from EQ-5D-3L responses observed at baseline and 6, 12, 18 and 24 months and the discounted QALY total was estimated as the area under the linear interpolation of the five points. We then repeated the QALY estimation using EQ-5D-5L predicted from the full mixture-copula model presented in Section 4.2, conditional on the demographic and clinical covariates and EQ-5D-3 responses observed in the trial. Note that, since this construction is a linear function of the EQ-5D responses *Y*, our use of *E*(*Y*_5_|*Y*_3_, *X*) as a predictor does not introduce bias into the QALY evaluation, as it would for a nonlinear function of *Y*.

**Table 7:** Mean costs, QALYs and incremental cost-effectiveness ratios for the CARDERA trial.

	Monotherapy	Combination therapies		
	MTX	MTX+CS	MTX+PNS	MTX+CS+PNS
Total costs^a^	£7503	£6829	£6323	£6203
**EQ-5D-3L from trial data**				
Total QALYs	1.238	1.093	1.152	1.320
ICER (*for col therapy vs. row therapy*)				
MTX only	–	£4648	£13,714	–£15,929
MTX+CS	£4648	–	–£8597	-£2765
MTX+PNS	£13,714	–£8597	–	–£714

**EQ-5D-5L mapped from 3L trial data (full joint copula-mixture model)**				
Total QALYs	1.450	1.351	1.382	1.513
*ICER (for col therapy vs. row therapy)*				
MTX only	–	£6,755	£17,264	–£20,728
MTX+CS	£6755	–	–£ 16,140	–£3,857
MTX+PNS	£17,264	–£ 16,140	–	–£917

**EQ-5D-5L mapped from 3L trial data for restricted models**				
*Demographic covariates only*				
Total QALYs	1.437	1.326	1.359	1.480
*ICER (for col therapy vs. row therapy)*				
MTX only	–	£6054	£15,137	–£30,466
MTX+CS	£6054	–	–£15,198	–£4070
MTX+PNS	£15,137	–£15,198	–	–£996

*Independent domains*				
Total QALYs	1.462	1.376	1.404	1.531
*ICER (for col therapy vs. row therapy)*				
MTX only	–	£7851	£20,361	–£18,696
MTX+CS	£7851	–	–£18,179	–£4033
MTX+PNS	£20,361	–£18,179	–	–£942

*Joint Gaussian model*				
Total QALYs	1.453	1.353	1.384	1.514
*ICER (for col therapy vs. row therapy)*				
MTX only	–	£6818	£17,409	–£20,708
MTX+CS	£6,818	–	–£16,324	–£3877
MTX+PNS	£17,409	–£16,324	–	–£920

a Present value of treatment costs over the 2-year experimental period.

The cost-effectiveness results are presented in the first two panels of Table 7.^8^ Of the four treatment strategies, triple therapy is the least costly and most effective, thus dominating all other strategies. Among the remaining three treatment strategies, the MTS+CS combination is dominated by MTX plus steroid, being more costly and less effective. Monotherapy is more costly but also more effective than MTX plus steroid, with an ICER of £13,714 which lies comfortably below a conventional cost-effectiveness threshold of £20,000 per QALY. The effect of mapping is to increase the estimated dominance of the triple therapy over all others and also the dominance of MTX+PNS over MTX+CS. The ICER for monotherapy versus MTX+PNS increases from £13,714 to £17,264, which remains below the conventional threshold. Thus, mapping has increased the magnitude of estimated ICERs, but without changing any of the decisions that would be likely to follow.

The mapped EQ-5D-5L QALYs are larger (by 15–24%) than the directly-measured EQ-5D-3L QALY estimates; but critically, they also vary less proportionately – the range of QALYs is 20% of the smallest for EQ-5L-3L but 12% for mapped EQ-5D-5L. Because the QALY is in the ICER denominator, the six ICERs for pairwise comparisons of the therapies increase in magnitude – by more than 100% in some cases. This result is partly due to the significant response differences to the mobility and pain questions, but also to the large negative values built into the Dolan (1997) utility scoring system which tends to increase the coefficient of variation of 3L scores relative to 5L scores. Thus a substantial part of the increase in ICERs when using mapping is attributable not to mapping *per se*, but to the different structures of the 3L and 5L scoring systems. This suggests that we can expect to see similar results if we adopt EQ-5D-5L in many other evaluation settings – perhaps warranting a future reassessment of the cost-effectiveness threshold by bodies such as NICE. Preliminary work by Hernández-Alava et al. (2017) tends to support this view.

We can explore the impact of mapping in the remainder of Table 7 by showing the effects on cost-effectiveness results of using three alternative simplified versions of the mapping model. It is common practice in economic evaluation to use very limited sets of covariates in mapping models; the first restricted model investigates this by dropping from the model the five (highly significant) covariates based on the HAQ and pain scale clinical measures. Simplifying the covariate list has the effect of greatly increasing the apparent dominance of the triple therapy over all others, with the ICER relative to monotherapy rising by almost 50% in magnitude. Again, it is unlikely that cost-effectiveness decisions would differ from those made with direct measurement of EQ-5D-3L.

The second simplified version of the mapping model retains the full set of covariates but imposes the restriction of independence across health domains by eliminating the random effect *V* through the parameter restrictions *ψ*_*kd*_ = 0, which are strongly rejected by direct tests. Relative to the full mapping model, most ICERs increase in magnitude under the independence restriction and, in the case of monotherapy versus the MTX/steroid combination, the increase takes the ICER beyond the £20,000 threshold, which would bring the cost-effectiveness of monotherapy into question in a comparison between the two. That ICER is almost 50% greater than the estimate derived from direct observation of EQ-5D-3L.

The third simplified model retains the full covariate vector and cross-domain correlation, but imposes normality on the error distributions by eliminating all mixture parameters and imposing the Gaussian copula in all of the five domains. Here the ICER results are similar to those of the full model and consequent cost-effectiveness decisions.

The differences between cost-effectiveness estimates derived from different versions of the mapping model are potentially large enough to alter policy decisions. For example, the ICER comparing monotherapy with combination DMARD + steroid rises by 18% from £17,264 to £20,361 when we switch to the independent domains model. If we were to use a cost-effectiveness threshold of £20,000, this would question the decision that monotherapy is cost-effective relative to the DMARD + steroid combination therapy. Using the joint model, the ICER rises to £17,264, not large enough to reverse the decision but a substantial rise nonetheless.^9^ Since the ICER is the ratio of a cost difference to a QALY difference, it is particularly sensitive to changes in the denominator when alternative treatments have similar impacts on QALYs.

## 7 Conclusions

There are three clear conclusions. First, econometric modelling based on a flexible mixture-copula specification has revealed significant differences between the 3L and 5L versions of the EQ-5D descriptive system for health states. These differences are particularly striking for the mobility and pain domains, where the two versions of the instrument give significantly different pictures of the relationship between individual health states and their demographic and clinical determinants.

Second, we have developed a new and powerful technique for modelling and mapping between the 3L and 5L health descriptions provided by the two variants of EQ-5D, using a conditional expectation approach. In this framework, we map between health descriptive systems before applying utility scores, and this mapping procedure reproduces the directly-observed distributional shape quite faithfully. On the basis of the evidence presented here, NICE could move to the new 5L version of EQ-5D as the basis for its decision-making, and use flexible mapping techniques where necessary to convert old 3L evidence to the new basis. The alternative approach of direct mapping between utility scores can reproduce distributional features accurately if a sufficiently flexible model is specified (Hernández-Alava et al., 2012), but that approach ignores the richer information available in the health descriptions *Y*_31_, …, *Y*_35_ and *Y*_51_, …, *Y*_55_ and does not allow comparisons to be made across domains. Perhaps most importantly, the direct approach conflates the effect of the redesigned health description and the revised utility tariff and does not offer a natural way of comparing alternative utility tariffs.

Third, our re-examination of evidence from a trial of combination drug therapies for rheumatoid arthritis shows that switching to the newer 5L version of EQ-5D and using the utility scoring system recently proposed by Devlin et al. (2016) can make a substantial difference to the conclusions from cost-effectiveness studies. This is partly a consequence of the different utility tariffs developed for EQ-5D-3L and EQ-5D-5L which itself may call for some adjustment to the way that such studies are translated into funding decisions. But, working within a comprehensive and flexible framework that models 3L and 5L jointly, we have shown that econometric specification can also have a separate large impact. In particular, making the simplifying assumption of independence across health domains, or using a restricted set of covariates that excludes clinical information, may cause large shifts in cost-effectiveness ratios – of up to 50% in our application to rheumatic disease.

## Figures and Tables

**Table A1:** Estimated coefficients of the domain-specific bivariate and joint models.

	Domain-specific model		Joint model	
	Coefficient	Std. error	Coefficient	Std. error
*Mobility domain – 3 levels*				
Male	0.4601	0.0543	0.5125	0.0637
Age/10	−0.0117	0.0169	−0.0067	0.0197
Pain/10	2.4178	0.3205	2.8928	0.3826
HAQ	1.2370	0.1092	1.3765	0.1347
HAQ^2^	−0.9591	0.3880	0.0987	0.0627
Pain^2^	0.0593	0.0522	−1.2067	0.4554
HAQ × pain	−0.3067	0.1603	−0.3134	0.1907
*ψ*			0.6494	0.0416
Γ_1_	1.8996	0.1244	2.2583	0.1547
Γ_2_	5.6557	0.1634	6.7752	0.2465

*Mobility domain – 5 levels*				
Male	0.3390	0.0430	0.3839	0.0504
Age/10	0.0506	0.0137	0.0612	0.0159
Pain/10	1.9446	0.2525	2.4359	0.2964
HAQ	1.2235	0.0841	1.4009	0.1010
HAQ^2^	−0.4122	0.3099	0.0610	0.0470
Pain^2^	0.0458	0.0397	−0.6556	0.3606
HAQ × pain	−0.3969	0.1283	−0.4656	0.1527
*ψ*			0.6279	0.0317
Γ_1_	1.5939	0.0982	1.8964	0.1184
Γ_2_	2.9367	0.1032	3.4302	0.1321
Γ_3_	4.2711	0.1093	4.9911	0.1511
Γ_4_	5.5625	0.1303	6.5589	0.1920
Dependency *θ*	0.7074	0.0139	0.5956	0.0203

*Self-care domain – 3 levels*				
Male	0.6103	0.0662	0.6438	0.0688
Age/10	−0.1067	0.0204	−0.1096	0.0210
Pain/10	1.0591	0.4462	1.4948	0.4722
HAQ	1.8555	0.1966	1.9641	0.2226
HAQ^2^	−0.6821	0.4457	−0.0444	0.0790
Pain^2^	−0.0314	0.0729	−1.0048	0.4603
HAQ × pain	0.0428	0.2036	0.0040	0.2144
*ψ*			0.3163	0.0347
Γ_1_	2.7358	0.1960	2.9350	0.2235
Γ_2_	5.7598	0.2142	6.1590	0.2565

*Self-care domain – 5 levels*				
Male	0.6366	0.0536	0.6779	0.0569
Age/10	−0.0949	0.0167	−0.1006	0.0175
Pain/10	1.2139	0.3390	1.7335	0.3669
HAQ	1.5870	0.1270	1.7245	0.1432
HAQ^2^	−0.7787	0.3644	0.0097	0.0561
Pain^2^	0.0182	0.0519	−1.1726	0.3852
HAQ × pain	0.0764	0.1583	0.0276	0.1686
*ψ*			0.3806	0.0289
Γ_1_	2.0816	0.1350	2.3131	0.1524
Γ_2_	3.4855	0.1399	3.7768	0.1627
Γ_3_	4.9402	0.1512	5.3745	0.1825
Γ_4_	5.6903	0.1729	6.3115	0.2176
Dependency *θ*	6.0530	0.3145	5.5022	0.3051

*Usual activities domain – 3 levels*				
Male	0.2409	0.0539	0.3278	0.0781
Age/10	−0.0582	0.0168	−0.0751	0.0240
Pain/10	2.6254	0.3175	4.1937	0.4879
HAQ	1.7515	0.1164	2.6488	0.1936
HAQ^2^	−1.3382	0.3756	−0.3058	0.0709
Pain^2^	−0.1891	0.0503	−2.1676	0.5438
HAQ × pain	0.0196	0.1594	−0.1170	0.2237
*ψ*			1.0333	0.0819
Γ_1_	1.7532	0.1278	2.7194	0.2159
Γ_2_	4.7465	0.1520	6.9414	0.3559

*Usual activities domain – 5 levels*				
Male	0.1923	0.0440	0.2462	0.0625
Age/10	−0.0751	0.0139	−0.0961	0.0195
Pain/10	2.4151	0.2616	3.7146	0.3862
HAQ	1.6059	0.0925	2.2971	0.1437
HAQ^2^	−1.3418	0.3149	−0.1997	0.0581
Pain^2^	−0.1386	0.0416	−2.0802	0.4497
HAQ × pain	0.0367	0.1325	−0.0395	0.1881
*ψ*			0.9943	0.0616
Γ_1_	1.0144	0.0997	1.5766	0.1490
Γ_2_	2.4708	0.1074	3.6049	0.1854
Γ_3_	3.9116	0.1188	5.6372	0.2345
Γ_4_	4.8488	0.1342	6.8882	0.2712
Dependency *θ*	0.5560	0.0172	0.1019	0.0541

*Common mixture*				
*π*	0.0621	0.0461		
1 − *π*	0.9379	0.0461		
*μ*_1_	0.2841	0.4314		
*μ*_2_	−0.0188	0.0217		
$\sigma_{1}^{2}$	3.0482	0.8537		
$\sigma_{2}^{2}$	0.8587	0.0665		

*Pain/discomfort domain – 3 levels*				
Male	0.1737	0.0472	0.2130	0.0562
Age/10	0.0332	0.0156	0.0274	0.0181
Pain/10	6.3976	0.4445	7.1520	0.4037
HAQ	0.6059	0.0908	0.7806	0.1046
HAQ^2^	−2.3849	0.4493	−0.1176	0.0551
Pain^2^	−0.1296	0.0488	−3.0418	0.4349
HAQ × pain	0.4015	0.1796	0.1717	0.1849
*ψ*			0.3705	0.0325
Γ_1_	0.8379	0.1132	0.9465	0.1241
Γ_2_	5.1633	0.1728	5.4769	0.1890
*π*	0.5871	0.0787		
1 − *π*	0.4129	0.0787		
*μ*_1_	−0.0936	0.0528		
*μ*_2_	0.1331	0.0771		
$\sigma_{1}^{2}$	0.2850	0.0824		
$\sigma_{2}^{2}$	1.9866	0.2359		

*Pain/discomfort domain – 5 levels*				
Male	0.1085	0.0424	0.1278	0.0484
Age/10	−0.0504	0.0137	−0.0605	0.0155
Pain/10	6.0189	0.2887	6.9250	0.3362
HAQ	0.6694	0.0819	0.7903	0.0936
HAQ^2^	−2.6218	0.3451	−0.1119	0.0460
Pain^2^	−0.1042	0.0402	−3.0565	0.3848
HAQ × pain	0.3632	0.1391	0.3352	0.1563
*ψ*			0.5364	0.0301
Γ_1_	−0.3351	0.0939	−0.3981	0.1061
Γ_2_	2.0121	0.1049	2.3200	0.1212
Γ_3_	4.1984	0.1174	4.7505	0.1437
Γ_4_	5.3824	0.1280	6.0899	0.1616
*π*	0.1075	0.0745		
1 − *π*	0.8925	0.0745		
*μ*_1_	0.1204	0.1985		
*μ*_2_	−0.0145	0.0195		
$\sigma_{1}^{2}$	2.6886	0.7068		
$\sigma_{2}^{2}$	0.7948	0.0830		
Dependency *θ*	1.7094	0.0474	1.5660	0.0452

*Anxiety/depression domain – 3 levels*				
Male	0.0387	0.0491	0.0469	0.0495
Age/10	−0.1350	0.0148	−0.1355	0.0152
Pain/10	1.2087	0.2829	1.3453	0.2894
HAQ	0.4322	0.0904	0.4549	0.0923
HAQ^2^	−0.2623	0.3495	−0.0663	0.0440
Pain^2^	−0.0580	0.0436	−0.4026	0.3550
HAQ × pain	0.1788	0.1471	0.1903	0.1478
*ψ*			0.3257	0.0259
Γ_1_	0.4435	0.1033	0.4901	0.1055
Γ_2_	2.2668	0.1086	2.3920	0.1164

*Anxiety/depression domain – 5 levels*				
Male	−0.0137	0.0453	−0.0071	0.0462
Age/10	−0.1456	0.0137	−0.1482	0.0142
Pain/10	1.2094	0.2554	1.3614	0.2640
HAQ	0.3731	0.0826	0.4139	0.0855
HAQ^2^	−0.4111	0.3179	−0.0526	0.0410
Pain^2^	−0.0387	0.0401	−0.5557	0.3251
HAQ × pain	0.2730	0.1354	0.2818	0.1377
*ψ*			0.3554	0.0240
Γ_1_	0.1154	0.0945	0.1625	0.0979
Γ_2_	1.0888	0.0953	1.1589	0.0999
Γ_3_	2.0811	0.0998	2.2051	0.1076
Γ_4_	2.6195	0.1098	2.8087	0.1227
Dependency *θ*	14.4849	0.5894	13.9413	0.5912

*Common mixture – joint model*				
*π*			0.0250	0.0127
1 − *π*			0.9750	0.0127
*μ*_1_			−0.5004	0.2528
*μ*_2_			0.0128	0.0072
$\sigma_{1}^{2}$			5.6660	1.6944
$\sigma_{2}^{2}$			0.8739	0.0286

## Footnotes

1 Including Belgium, Colombia, Egypt, Estonia, Ireland, Latvia, Lithuania, the Netherlands, New Zealand and Sweden. See the pharmacoeconmics guidelines maintained by the International Society for Pharmacoeconomics and Outcomes Research (https://www.ispor.org/PEguidelines/COMP3.asp).

2 See the EuroQol website http://www.euroqol.org/eq-5d-products/how-to-obtain-eq-5d.html for examples of the question wording used in EQ-5D-3L and EQ-5D-5L.

3 A minority of cases come by self-referral, with medical details obtained by NDBRB in the same way.

4 Samples generated by Monte Carlo simulation, from copulas specified with Kendall's *τ* ≈ 0.7.

5 Note that these are formally tests of the hypothesis that the coefficient vectors are equal after each error variance is normalised to unity. Since the extreme points on the 3L and 5L scales are (mostly) given the same verbal labels to act as anchors, the assumption seems reasonable. Also, where differences are statistically significant, the 3L and 5L coefficient vectors are clearly not scalar multiples of each other.

6 Hernández-Alava and Pudney (2017) provide a Stata command eq5dmap that implements variants of this mapping operation.

7 Initially dosed at 60 mg/day, reducing to 7.5 mg/day at 6 weeks and stopped by 34 weeks.

8 Note that there are minor differences between the numbers reported in Table 7 and those in Wailoo et al. (2014) due to missing data in the variables used to predict EQ-5D-5L for one patient, but results are unaffected.

9 The first published version of the value set (Devlin and van Hout, 2015) produced higher ICERs, £21,476 and £18,100 respectively.

## References

[bib0005] Agborsangaya C.B., Lahtinen M., Cooke T., Johnson J.A. (2014). Comparing the EQ-5D 3L and 5L: measurement properties and association with chronic conditions and multimorbidity in the general population. Health Qual. Life Outcomes.

[bib0010] Augustovski F., Rey-Ares L., Irazola V., Garay O.U., Gianneo O., Fernández G., Morales M., Gibbons L., Ramos-Go ni J.M. (2016). An EQ-5D-5L value set based on Uruguayan population preferences?. Qual. Life Res..

[bib0015] Bedford T., Cooke R. (2002). Vines – a new graphical model for dependent random variables. Ann. Stat..

[bib0020] Brazier J., Tsuchiya A. (2015). Improving cross-sector comparisons: going beyond the health-related QALY. Appl. Health Econ. Health Policy.

[bib0025] Brazier J.E., Yang Y., Tsuchiya A., Rowen D.L. (2010). A review of studies mapping (or cross walking) non-preference based measures of health to generic preference-based measures?. Eur. J. Health Economics.

[bib0030] Bruce B., Fries J.F. (2003). The Stanford Health Assessment Questionnaire (HAQ): a review of its history, issues, progress, and documentation. J. Rheumatol..

[bib0035] Choy E.H.S., Smith C.M., Farewell V., Walker D., Hassell A., Chau L., Scott D.L. (2008). Factorial randomised controlled trial of glucocorticoids and combination disease modifying drugs in early rheumatoid arthritis. Ann. Rheum. Dis..

[bib0040] Claxton K., Martin S., Rice N., Spackman E., Hinde S., Devlin N., Smith P.C., Sculpher M. (2015). Methods for the estimation of the National Institute for Health and Care Excellence cost-effectiveness threshold. Health Technol. Assess..

[bib0045] Conigliani C., Manca A., Tancredi A. (2015). Prediction of patient-reported outcome measures via multivariate ordered probit models?. J. R. Stat. Soc. Ser. A (Stat. Soc.).

[bib0050] Cross M., Smith E., Hoy D., Carmona L., Wolfe F., Vos T., Williams B., Gabriel S., Lassere M., Johns N., Buchbinder R., Woolf A., March L. (2014). The global burden of rheumatoid arthritis: estimates from the Global Burden of Disease 2010 study?. Ann. Rheum. Dis..

[bib0055] Devlin N., Brooks R. (2017). EQ-5D and the EuroQol group: past, present and future. Appl. Health Econ. Health Policy.

[bib0060] Devlin N., Shah K., Feng Y., Mulhern B., van Hout B. (2016). Valuing health-related quality of life: an EQ-5D-5L value set for England. Technical Report 16.02.

[bib0065] Devlin N., van Hout B. (2015). An EQ-5D-5L Value Set for England.

[bib0070] Dolan P. (1997). Modeling valuations for EuroQol health states. Med. Care.

[bib0075] Fayers P.M., Hays R.D. (2014). Should linking replace regression when mapping from profile-based measures to preference-based measures?. Value Health.

[bib0080] Feng Y., Devlin N., Shah K., Mulhern B., van Hout B. (2016). New methods for modelling EQ-5D-5L value sets: an application to English data. Technical Report 16.03.

[bib0085] Gray A.M., Rivero-Arias O., Clarke P.M. (2006). Estimating the association between SF-12 responses and EQ-5D utility values by response mapping?. Med. Decis. Mak..

[bib0090] Hernández-Alava M., Pudney S.E. (2016). BICOP: a command for estimating bivariate ordinal regressions with residual dependence characterized by a copula function and normal mixture marginal?. Stata J..

[bib0095] Hernández-Alava M., Pudney S.E. (2017). eq5dmap: a Stata command for mapping from 3-level to 5-level EQ-5D. Technical report.

[bib0100] Hernández-Alava M., Wailoo A.J., Ara R. (2012). Tails from the peak district: adjusted limited dependent variable mixture models of EQ-5D health state utility values. Value Health.

[bib0105] Hernández-Alava M., Wailoo A.J., Grimm S., Pudney S.E., Gomes M., Sadique Z., Meads D., O’Dwyer J., Barton G., Irvine L. (2017). EQ-5D versus 3L: the impact on cost-effectiveness. Technical Report 17.02.

[bib0110] Ikeda S., Shiroiwa T., Igarashi A., Noto S., Fukuda T., Saito S., Shimozuma K. (2015). Developing a Japanese version of the EQ-5D-5L value set?. J. Natl. Inst. Public Health.

[bib0115] Janssen M.F., Pickard A.S., Golicki D., Gudex C., Niewada M., Scalone L., Swinburn P., Busschbach J. (2013). Measurement properties of the EQ-5D-5L compared to the EQ-5D-3L across eight patient groups: a multi-country study. Qual. Life Res..

[bib0120] Jia Y.X., Cui F.Q., Li L., Zhang D.L., Zhang G.M., Wang F.Z., Gong X.H., Zheng H., Wu Z.H., Miao N., Sun X.J., Zhang L., Lv J.J., Yang F. (2014). Comparison between the EQ-5D-5L and the EQ-5D-3L in patients with hepatitis B. Qual. Life Res..

[bib0125] Kim S.-H., Ahn J., Ock M., Shin S., Park J., Luo N., Jo M.-W. (2016). The EQ-5D-5L valuation study in Korea?. Qual. Life Res..

[bib0130] Longworth L., Rowen D. (2011). The use of mapping methods to estimate health state utility values. Technical Report NICE DSU Technical Support Document 10, Decision Support Unit.

[bib0135] Murray C.J.L.e. (2012). Disability-adjusted life years (DALYs) for 291 diseases and injuries in 21 regions, 1990–2010: a systematic analysis for the Global Burden of Disease Study 2010. Lancet.

[bib0140] NICE (2013). Guide to the methods of technology appraisal 2013. Technical report.

[bib0145] Panagiotelis A., Czado C., Joe H. (2012). Pair copula constructions for multivariate discrete data. J. Am. Stat. Assoc..

[bib0150] Pickard A.S., Leon M.C.D., Kohlmann T., Cella D., Rosenbloom S. (2007). Psychometric comparison of the standard EQ-5D to a 5 level version in cancer patients. Med. Care.

[bib0155] Scalone L., Ciampichini R., Fagiuoli S., Gardini I., Fusco F., Gaeta L., Prete A.D., Cesana G., Mantovani L.G. (2013). Comparing the performance of the standard EQ-5D 3L with the new version EQ-5D 5L in patients with chronic hepatic diseases. Qual. Life Res..

[bib0160] Trivedi P.K., Zimmer D.M. (2005). Copula modeling: an introduction for practitioners. Found. Trends Econom..

[bib0165] van Hout B., Janssen M.F., Feng Y.S., Kohlmann T., Busschbach J., Golicki D., Lloyd A., Scalone L., Kind P., Pickard A.S. (2012). Interim scoring for the EQ-5D-5L: mapping the EQ-5D-5L to EQ-5D-3L value sets. Value Health.

[bib0170] Versteegh M.M., Vermeulen K.M., Evers S.M.A.A., Ardine de Wit G., Prenger R., Stolk E.A. (2016). Dutch tariff for the five-level version of EQ-5D. Value Health.

[bib0175] Wailoo A., Hernández-Alava M., Scott I.C., Ibrahim F., Scott D.L. (2014). Cost-effectiveness of treatment strategies using combination disease-modifying anti-rheumatic drugs and glucocorticoids in early rheumatoid arthritis. Rheumatology.

[bib0180] Wolfe F., Michaud K. (2011). The National Data Bank for rheumatic diseases: a multi-registry rheumatic disease data bank. Rheumatology.

[bib0185] Xie F., Pullenayegum E., Gaebel K., Bansback N., Bryan S., Ohinmaa A., Poissant L., Johnson J.A. (2016). A time trade-off-derived value set of the EQ-5D-5L for Canada?. Med. Care.

